# Evaluation of Oil-Absorbing Film for Imprint Desorption Electrospray Ionization Mass Spectrometry Imaging (IDESI-MSI) on Biological Samples

**DOI:** 10.3390/metabo14030160

**Published:** 2024-03-11

**Authors:** Jiedong Li, Ruolun Wei, Yifan Meng, Richard N. Zare

**Affiliations:** 1 Department of Chemistry and Biochemistry, University of California, Santa Barbara, Santa Barbara, CA 93106, USA; jiedongli@ucsb.edu; 2 Department of Neurosurgery, Stanford University School of Medicine, Stanford, CA 94305, USA; rlwei@stanford.edu; 3 Department of Chemistry, Stanford University, Stanford, CA 94305, USA

**Keywords:** imprint DESI-MSI, oil-absorbing film, molecular imaging

## Abstract

Imprint Desorption Electrospray Ionization Mass Spectrometry Imaging (IDESI-MSI) has proven to be a robust and reliable tool for chemically imaging biological samples such as fungi, animal tissues, and plants, but the choice of the imprint substrate is crucial. It must effectively transfer maximum amounts of species from the sample while preserving the original spatial distribution of detected molecules. In this study, we explored the potential of utilizing an oil-absorbing film, known for its soft nature and excellent lipophilicity, as an imprint substrate for IDESI-MSI on biological samples. To assess the transfer efficiency of the amounts of molecules and molecular patterns, we conducted experiments using mouse brain tissue. The result shows that more than 90% of the analytes can be transferred to the oil-absorbing film from the original tissue. A comparison of IDESI-MSI results between the oil-absorbing film and the original tissue demonstrates the material’s capability to transfer most molecules from the original tissue and retain images of different analytes with high spatial fidelity. We extended our investigation to plant imaging, where we applied IDESI-MSI to a cross-section of okra. The oil-absorbing film exhibited promise in this context as well. These findings suggest that IDESI-MSI utilizing the oil-absorbing film holds potential across various research fields, including biological metabolism, chemistry, and clinical research, making this technique widely applicable.

## 1. Introduction

Mass spectrometry imaging (MSI) is a surface analysis technique to visualize the spatial distribution of molecules according to their mass-to-charge ratio (*m*/*z*) [1,2,3]. By using different sampling and ionization techniques, various MSI approaches were developed, such as laser desorption/ionization mass spectrometry (LDI-MS) [4,5], matrix-assisted laser desorption/ionization mass spectrometry (MALDI-MS) [6,7], laser ablation electrospray ionization mass spectrometry (LA-ESI-MS) [8,9], laser ablation inductively coupled plasma mass spectrometry (LA-ICP-MS) [10,11], secondary-ion mass spectrometry (SIMS) [12,13], desorption electrospray ionization mass spectrometry (DESI-MS) [14,15,16,17], and liquid extraction surface analysis (LESA) techniques [18].

Without the requirement of sampling and ionization in a high vacuum environment, DESI-MS, which is an ambient MS method that operates in atmospheric pressure and temperature, is widely used in various fields, including chemistry [19], biology [20,21], and medical studies [22]. In DESI, high-speed charged microdroplets are sprayed by nebulizing gas against the sample surface and induce the sample to be charged and desorbed into the air and analyzed by the mass spectrometer. With a software-controlled stage, the sample moves automatically, and the detected *m*/*z* value corresponds to a specific molecule in the probed sample location. 

For biological applications, DESI-MSI has a critical requirement for sample preparation. Normally, biological tissues should be cut into thin sections (normally 10–20 μm thickness) with a freezing microtome, which brings time and economic cost [23]. Recently, imprint techniques were developed to avoid the complex sample preparation of MSI [24,25]. By extracting the analytes from the sample surface to a transfer material, molecules can be detected from the imprint rather than the original sample surface. For this purpose, the imprint material is required to transfer as much of the species as possible from the sample surface and retain the original distribution of detected molecules. Previously, the imprint desorption electrospray ionization mass spectrometry imaging (IDESI-MSI) techniques were reported to be successfully applied on various materials, such as thin layer chromatography (TLC) surface [26], tape [27], polytetrafluoroethylene (PTFE) [24,26], and porous silicon (pSi) [26]. In this study, we developed a new material, an oil-absorbing film, for IDESI-MSI on biological samples. We evaluated the transfer efficiency of the oil-absorbing film by quantitatively comparing the peaks from the mass spectrum with peaks from the original sample. Also, we explored the ability of this imprint material to transfer molecular patterns on mouse brain tissue and fresh plant samples. Results indicate that the oil-absorbing film can be used as an imprint material in DESI-MSI because it can extract most of the molecules from the sample surface and keep the original chemical distribution.

## 2. Materials and Methods

### 2.1. Chemicals and Reagents

A solution of acetonitrile (ACN) and N, N-dimethylformamide (DMF) (*v*:*v* = 1:1) was used as the DESI solvent. All the chemicals were purchased from Sigma-Aldrich (St. Louis, MO, USA).

### 2.2. Oil-Absorbing Film

The oil-absorbing film was purchased from Johnson & Johnson (Brand: Clean & Clear, New Brunswick, NJ, USA), which was originally used as an external-use material to remove excessive sebum from human skin. The film consists of polypropylene and mineral oil [28]. As a commercial product, this film is non-toxic and is considered to be a skin-friendly material. 

### 2.3. Sample Preparation

Mouse brain section. Nsg mice were anesthetized before being decapitated. The brain tissue was dissected and embedded in OCT and frozen at −80 °C. The mouse brain tissue sections of 12 μm thickness were obtained by a cryostat (Leica CM1900, Welzlar, Germany). Sections were mounted on glass slides. Previous research demonstrated that there was no significant difference between mass spectrum quality and number of lipids identified in biological tissue under negative ion mode. MSI of OCT-embedded tissues was compatible with negative ion mode imaging [29,30]. Because all MS data are collected in negative ion mode, we did not wash the mouse brain section to remove polymers from OCT or use other MSI-friendly embedding material in the section preparation process.

Imprint of mouse brain. The adjacent surface, after sectioning of mouse brain tissue, was used to prepare the imprint sample. An oil-absorbing film (Clean & Clear, New Brunswick, NJ, USA) was pressed against the mouse brain cross-section for 10 s to transfer analytes from the tissue. Then, the film-extracted samples from mouse brain were mounted on the DESI-MSI platform for imaging experiment. 

Imprint of okra. Fresh okra was purchased from a local grocery store in Palo Alto, CA, USA. Cut the okra with a knife to obtain a fresh cross-section. The oil-absorbing film was pressed against the okra cross-section for 10 s to transfer analytes from the sample surface. Then, the film containing analytes was mounted on the DESI-MSI platform.

### 2.4. DESI-MSI

MSI experiments were carried out with a commercial DESI source (Prosolia Inc., Indianapolis, IN, USA). A commercial DESI sprayer (Viktor Technology Co., Ltd., Beijing, China) was used in this experiment. The inner diameter (i.d.) of the DESI spray capillary was 20 μm, and the outer diameter (o.d.) of the capillary was 120 μm. During imaging process, the Y-distance between the DESI tip and the sample surface was set as 4 mm. The X-distance between the DESI tip and the MS inlet was set as 2 mm. The impact angle between the DESI sprayer and the sample stage was set at 60°. Compressed N_2_ (120 psi, 99.999% purity) was used as the nebulizing gas. A negative voltage (−6 kV) was applied to the DESI sprayer. All mass spectra were recorded in negative ion mode. The injection rate of DESI solvent (ACN:DMF = 1:1) was set as 0.7 μL/min. During the data-collecting process, the moving speed of the X-axis of the sample platform was set as 300 μm/s, while the step size of the Y-axis was set as 80 μm. The spatial resolution of DESI-MSI is about 100 microns. An Orbitrap mass spectrometer (Velos Pro, Thermo Fisher Scientific, Waltham, MA, USA) was used to obtain the MS data. Negative full MS scans were employed over the range of *m*/*z* 200–1000. The automatic gain control (AGC) was set to the off position to keep the scan rate constant. The mass resolution was set to 30,000. The maximum injection time was set as 100 μs. The MS inlet capillary temperature was set at 300 °C. MS images are generated using a self-coded program running in MATLAB (2021b, MathWorks, Natick, MA, USA).

## 3. Results

### 3.1. Comparison of IDESI-MSI on an Oil-Absorbing Film and Conventional DESI-MSI on a Mouse Brain Section

**Figure 1:**
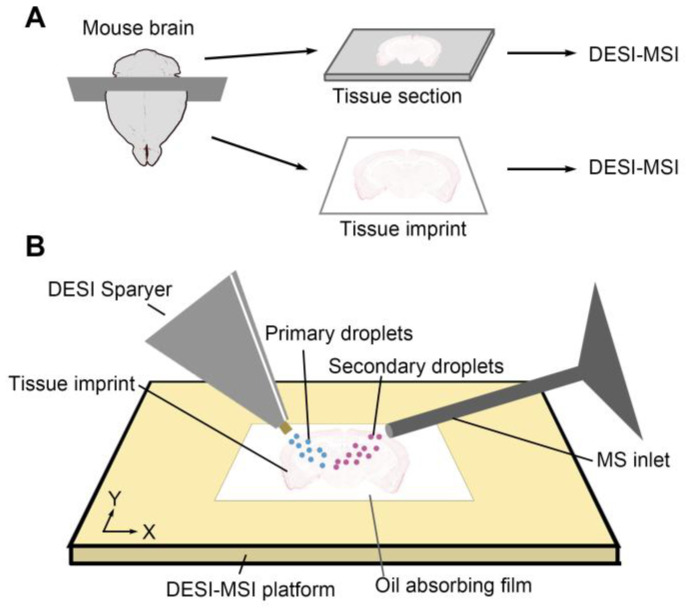
Schematic diagram of the IDESI-MSI experiment with an oil-absorbing film. (**A**) Sample preparation for mouse brain tissue. (**B**) IDESI-MSI setup.

To evaluate the ability of the oil-absorbing film in the imprint application, we first compared the IDESI-MSI with standard DESI-MSI on a mouse brain tissue sample. As shown in Figure 1A, a mouse brain tissue was frozen and cut into. The section was mounted onto a glass slide for further imaging experiments. This tissue section was used as a standard comparison while evaluating the ability of oil-absorbing film in imprint imaging. For the imprint experiment, the oil-absorbing film was pressed against the rest of the tissue bulk. So, the MS images on the tissue section and the imprint can be considered to be performed on the same surface. In this process, the oil-absorbing film was applied to the tissue surface with uniform pressure to obtain better contact extraction of the analytes from the tissue. Then, the tissue section and oil-absorbing film containing the tissue imprint were placed on the sample stage with the imprinted aspect oriented up towards the spray capillary. The MSI experiment setup is shown in Figure 1B. Primary charged solvent droplets were generated by the DESI emitter and applied on the imprinted tape surface. Then, secondary droplets containing analytes were directly detected by the mass spectrometer. 

**Figure 2:**
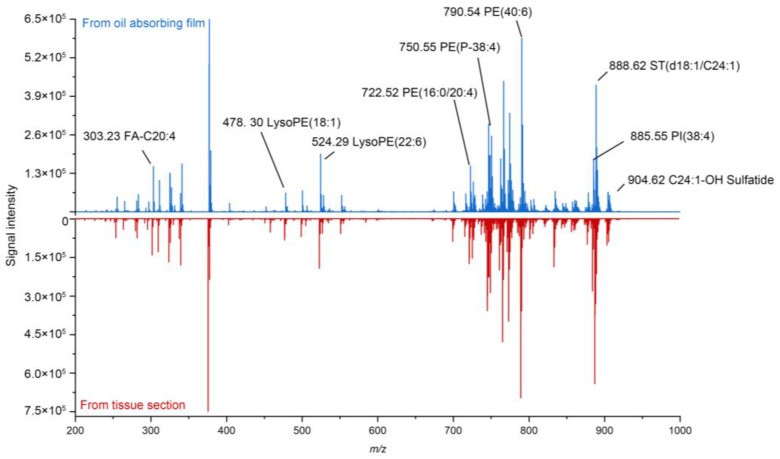
Comparison of mass spectra obtained from the imprint of mouse brain tissue on the oil-absorbing film and from original tissue section. Data were collected in negative ion mode.

Figure 2 shows a typical mass spectrum obtained from the imprint of mouse brain tissue on the oil-absorbing film. We can observe from the mass spectrum that various analytes can be detected from the imprint, including small molecule metabolites, fatty acids, and lipids. For example, the peak at *m*/*z* 303.23 represents FA-C20:4, which is an abundant fatty acid in brain tissue. The peak at *m*/*z* 478.30 represents LysoPE(18:1), and the peak at *m*/*z* 524.29 represents LysoPE(22:6), which are two main components of the cell membrane [31]. Also, multiple lipids can be detected from the IDESI-MSI technique, such as PE(16:0/20:4) at *m*/*z* 722.52, PE(P-38:4) at *m*/*z* 750.55, PE(40:6) at *m*/*z* 790.54, PI(38:4) at *m*/*z* 885.55, ST(d18:1/C24:1) at *m*/*z* 888.62, and C24:1-OH Sulfatide at *m*/*z* 904.62. Overall, the average signal intensity of the ions on the oil-absorbing film is about 90% of that on the original section. Table 1 shows the list of abundant ions that can be detected from the oil-absorbing film. The compared mass spectra of blank imprinting material and tissue imprint from *m*/*z* 100 to 1000 are shown in Supporting Information (Figure S1). We also compared the imprint efficiency of the oil-absorbing film and a conventional material, a TLC plate (Figure S2). By imprinting mouse brain tissue to these two materials, the mass spectrum of the analytes from a mouse brain can be detected by DESI. The result demonstrates that the oil-absorbing film has a higher imprint efficiency for lipids. Also, the total ion intensity is higher on the oil-absorbing film.

**Figure 3:**
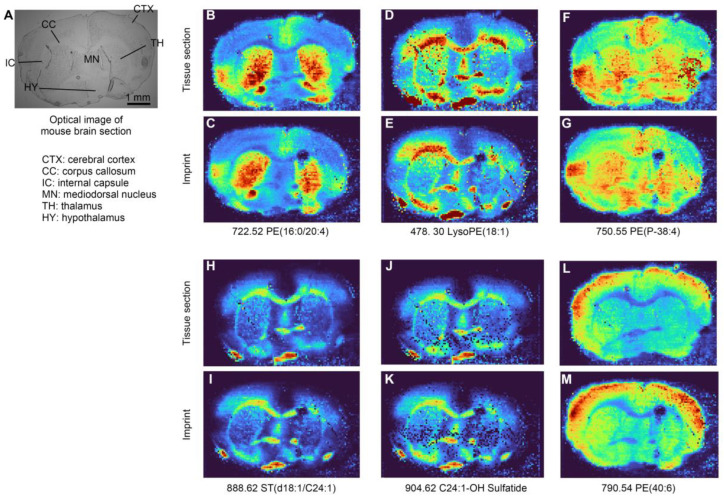
Compared DESI-MSI results from original tissue section and imprint on the oil-absorbing film. (**A**) Optical image of the mouse brain section. (**B**,**C**) MS images of PE(16:0/20:4) at *m*/*z* 722.52 from tissue section (**top**) and the imprint (**bottom**). (**D**,**E**) MS images of LysoPE(18:1) at *m*/*z* 478.30 from tissue section and the imprint. (**F**,**G**) MS images of PE(P-38:4) at *m*/*z* 750.55 from tissue section and the imprint. (**H**,**I**) MS images of ST(d18:1/C24:1) at *m*/*z* 888.62. (**J**,**K**) MS images of C24:1-OH Sulfatide at *m*/*z* 904.62 from tissue section and the imprint. (**L**,**M**) PE(40:6) at *m*/*z* 790.52 from original tissue and the imprint.

In addition to the number of analytes that can be transferred, another factor to evaluate the working ability of the oil-absorbing film is the imprint of MS images of different ions. We use MS images obtained by DESI-MSI from the original mouse brain section as the reference standard and evaluate the imprint accuracy of the molecular patterns by comparing them with MS images collected from the oil-absorbing film. Figure 3 shows the compared MSI results of six molecules from the original tissue section and the imprint, including PE(16:0/20:4) at *m*/*z* 722.52, LysoPE(18:1) at *m*/*z* 478.30, PE(P-38:4) at *m*/*z* 750.55, ST(d18:1/C24:1) at *m*/*z* 888.62, C24:1-OH Sulfatide at *m*/*z* 904.62, and PE(40:6) at *m*/*z* 790.52. The results indicate that the pattern of the same molecule from the oil-absorbing film shows a high consistency with it from the standard reference (tissue section). The optical image of the mouse brain section is shown in Figure 3A, in which several areas are identified, including the cerebral cortex (CTX), corpus callosum (CC), internal capsule (IC), mediodorsal nucleus (MN), thalamus (TH), and hypothalamus (HY). Figure 3B,C,F,G demonstrate the distribution of PE(16:0/20:4) and PE(P-38:4) in the original section and the oil-absorbing film, which have a higher concentration in TH. The relative abundance of LysoPE(18:1), ST(d18:1/C24:1), and C24:1-OH Sulfatide is higher in CC, IC, and HY, which are shown in Figure 3D,E,H–K. PE(40:6) has a higher concentration in the CTX area, which is shown in Figure 3L,M. These results demonstrate that not only the distribution of analytes but also the relative concentration can be transferred to the oil-absorbing film from the original tissue. The IDESI-MSI based on this material shows a potential to reveal the chemical distribution of multiple analytes without complicated sample preparation processes. 

### 3.2. IDESI-MSI of Fresh Okra Section

**Figure 4:**
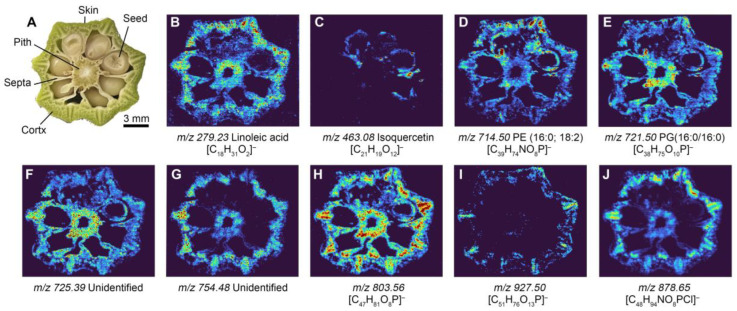
IDESI-MSI of fresh okra sample. (**A**) Optical image of the okra section. (**B**–**J**) IDESI-MSI images of selected ions at *m*/*z* 279.23, 463.08, 714.50, 721.50, 725.39, 754.48, 803.56, 927.50, and 878.65, respectively.

Furthermore, we also explored the possibility of using IDESI-MSI on plant samples. Okra from the local market was used as a sample. To determine the chemical composition of the okra section, we cut the okra and obtained a fresh surface. Then, the oil-absorbing film was applied against the surface for 5 s to extract the analytes from the okra section. Figure 4A shows the optical image of the okra section, which contains skin, cortex, septum, pitch, and seed. Figure 4B to Figure 4J shows the IDESI-MSI image of selected ions at *m*/*z* 279.23, 463.08, 714.50, 721.50, 725.39, 754.48, 803.56, 927.50, and 878.65, respectively. These images represent the distribution of different molecules in the okra section. It can be seen that not only some small molecules can be detected, such as FA(18:2) at *m*/*z* 279.23, but some lipid molecules are also detectable. The molecular patterns of different ions are clear. For example, the ions at *m*/*z* 279.23, 721.50, 725.39, 803.56, and 878.65 are mainly located in the cortex, septum, and pith (Figure 4B,E,F,H,J). The ion at *m*/*z* 463.08 only appears in the skin of the seed (Figure 4C). The ion at *m*/*z* 754.48 is mainly located in the cortex and pith (Figure 4G). And the selected ion at *m*/*z* 927.50 is only located in the skin of okra (Figure 4I). These results demonstrate the potential of exploring the chemical distribution in plant samples with IDESI-MSI using an oil-absorbing film. With this technique, we can visualize as many molecules as needed and shorten the time required for sample preparation. 

## 4. Conclusions

For applications of IDESI-MSI, the imprint substrate is required to transfer as many analytes as possible from the sample and retain the original distribution of detected molecules. In this study, we evaluated the potential use of an oil-absorbing film for IDESI-MSI. A mouse brain tissue is used to test the transfer ability for molecular amounts and chemical images of the film. The comparison results of mass spectra from the oil-absorbing film and the original tissue indicate that this material can transfer most molecules from the original tissue, including metabolites, fatty acids, and lipids. In addition, the molecular images from the oil-absorbing film are highly consistent with the images from the original tissue. This result indicates that the film can maintain the chemical distribution of different analytes during the imprint process. We also test the ability of this material in the IDESI-MSI of plant samples with a fresh okra section. All the results demonstrate that the oil-absorbing film can be used as an imprint material for DESI-MSI in biological samples without complex sample preparation. Considering that the oil-absorbing film consists of polypropylene, it has a good affinity for organics. The transfer efficiency of lipids is higher while using oil-absorbing film rather than other materials.

Compared with conventional DESI-MSI on a flat tissue section surface, IDESI-MSI on the oil-absorbing film requires more careful operations on sample preparation. For example, no relative displacement between the tissue and the oil-absorbing film should occur to ensure the accuracy of the imprint MS image. When mounting the soft film onto the DESI moving stage, it should be flat enough to keep the signal stable. In addition, for irregularly shaped samples, the force should be uniform during the imprinting process. These limitations should be considered when using this technique.

## Figures and Tables

**Table 1:** Some of the abundant ions that can be detected from oil-absorbing film.

Measured *m*/*z*	Theoretical *m*/*z*	Attribution
124.01	124.0068	Taurine
224.03		Not determined
255.23	255.2329	Palmitic acid
279.23	279.2330	FA(18:2)
281.23	281.2486	Oleic acid
283.26	283.2643	FA(18:0)
303.23	303.2329	FA(20:4)
311.16	311.1686	N-Undecylbenzenesulfonic acid
327.23	327.2330	FA(22:6)
465.31	465.3044	Cholesterol sulfate
478.30	478.2928	LysoPE(18:1)
524.29	524.2782	LysoPE(22:6)
722.52	722.5119	PE(16:0/20:4)
750.55	750.5432	PE(P-38:4)
790.54	790.5381	PE(40:6)
885.55	885.5487	PI(38:4)
888.62	888.6229	ST(d18:1/C24:1)
904.62	904.6178	C24:1-OH Sulfatide

## Data Availability

Data are available upon reasonable request from the corresponding authors. The data are not publicly available due to privacy and ethical restrictions.

## Supplementary Materials

The following supporting information can be downloaded at: https://www.mdpi.com/article/10.3390/metabo14030160/s1, Figure S1: Compared mass spectrum of blank imprinting material and tissue imprint from *m/z* 100 to 1000, Figure S2: Compared mass spectrum of mouse brain tissue imprint on (A) oil absorbing film and (B) TLC plate.
