# Supplementary material for: Evaluation of Oil-Absorbing Film for Imprint Desorption Electrospray Ionization Mass Spectrometry Imaging (IDESI-MSI) on Biological Samples
Source: Metabolites. 2024 Mar 11;14(3):160. doi: 10.3390/metabo14030160 (PMC10972117; doi:10.3390/metabo14030160)
Supplement: Supplementary file 1 [file metabolites-14-00160-s001.zip › metabolites-2873115-supplementary.pdf]

Supporting Information for

Evaluation of oil absorbing film for imprint desorption electrospray ionization mass spectrometry imaging (IDESI-MSI) on biological samples

Jiedong Li [1], Ruolun Wei [2], Yifan Meng [3,*] and Richard N. Zare [3,*]

[1] Department of Chemistry and Biochemistry, University of California, Santa Barbara, Santa Barbara, CA 93106
[2] Department of Neurosurgery, Stanford University School of Medicine, Stanford, CA, 94305
[3] Department of Chemistry, Stanford University, Stanford, CA, 94305
* Correspondence: Email: zare@stanford.edu; yfmeng@stanford.edu

Contents

Figure S1. Compared mass spectrum of blank imprinting material and tissue imprint. p S3

Figure S2. Compared mass spectrum of mouse brain tissue imprint on oil absorbing film and TLC plate. p S4

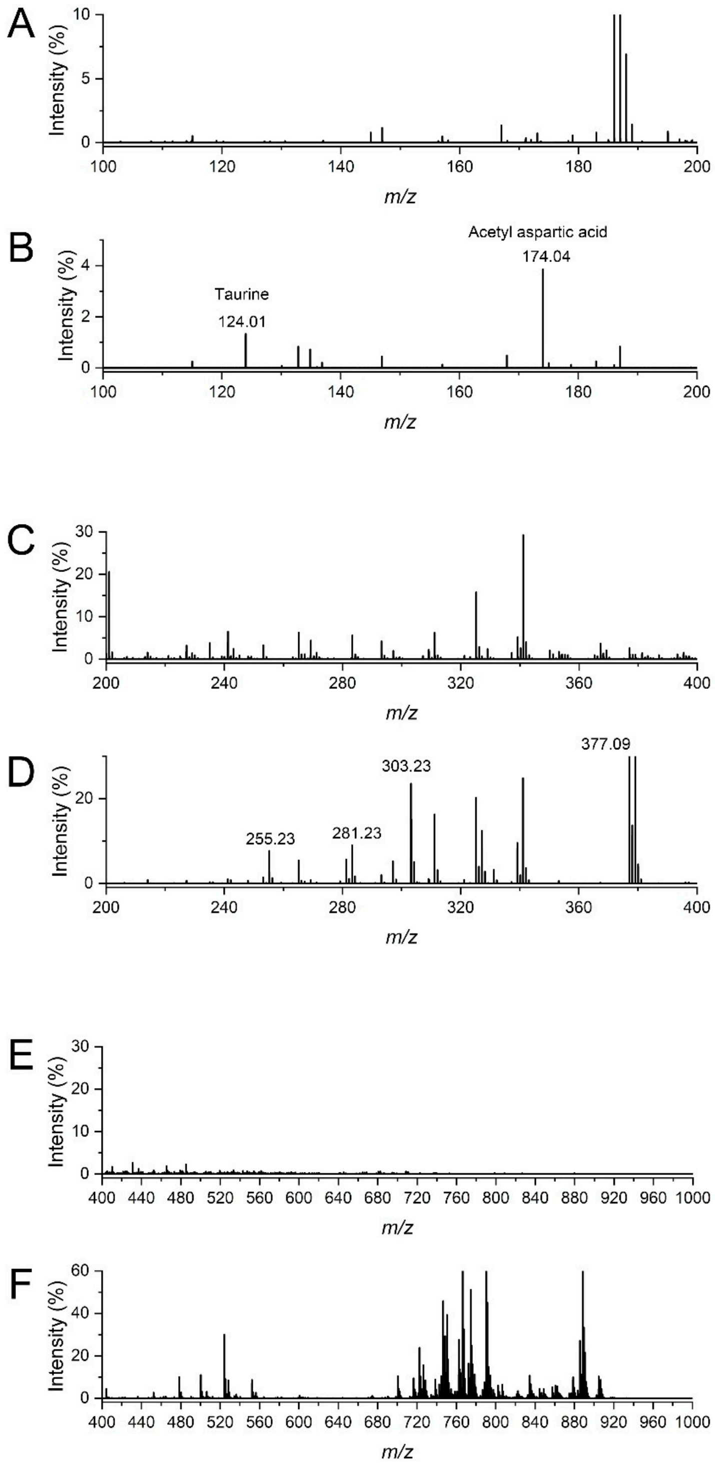

Figure S1. Compared mass spectrum of blank imprinting material and tissue imprint from *m/z* 100 to 1000. The full mass spectrum is separated to three ranges to display. Figures A, C, and E are mass spectra from blank imprinting material. Figure B, D, and F are mass spectra from mouse brain tissue imprint.

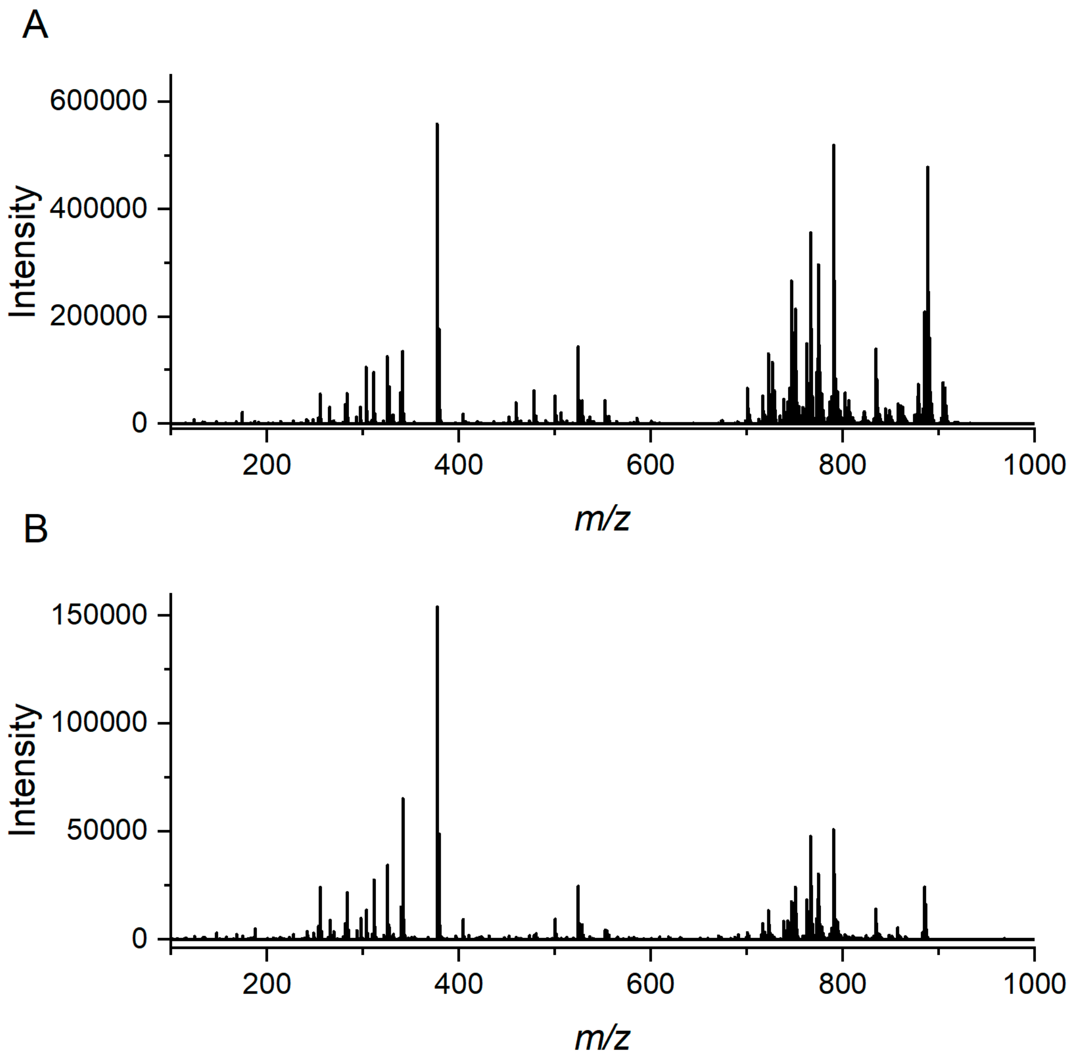

Figure S2. Compared mass spectrum of mouse brain tissue imprint on (A) oil absorbing film and (B) TLC plate.
